# Supplementary material for: Quality of cowpea seeds: A food security strategy in the tropical environment
Source: PLoS One. 2022 Oct 14;17(10):e0276136. doi: 10.1371/journal.pone.0276136 (PMC9565620; doi:10.1371/journal.pone.0276136)
Supplement: S1 Fig — (DOCX) [file pone.0276136.s001.docx]

**
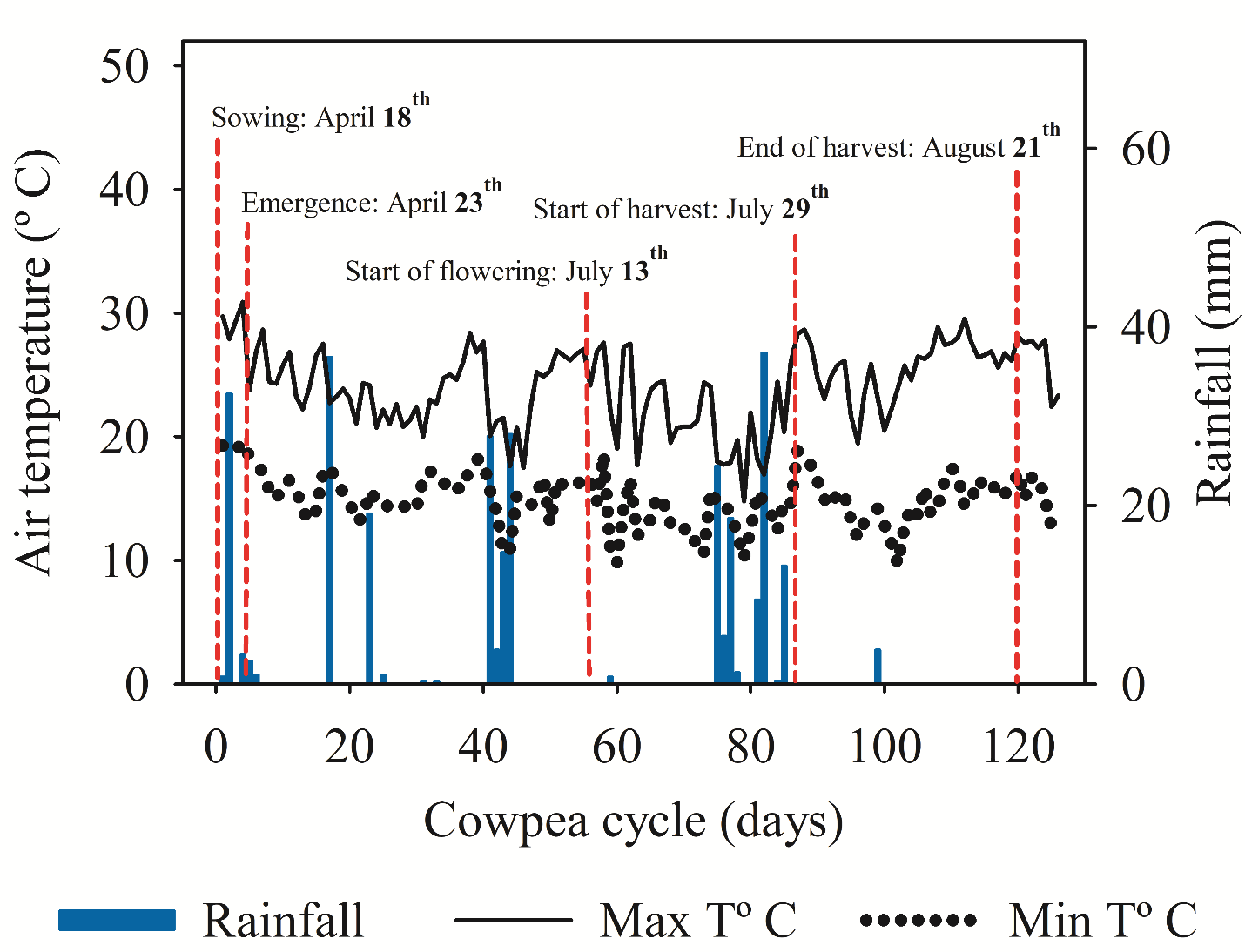
**

**S1 Fig.** Daily rainfall, relative air humidity, maximum and minimum temperatures in the experimental farm of Botucatu, State of São Paulo-Brazil, during cowpea seed production (2015 crop season).
