# Supplementary material for: Quality of cowpea seeds: A food security strategy in the tropical environment
Source: PLoS One. 2022 Oct 14;17(10):e0276136. doi: 10.1371/journal.pone.0276136 (PMC9565620; doi:10.1371/journal.pone.0276136)
Supplement: S2 Fig — A: Open flower; B: Flower after 2 days of pollination; C: Fruit in formation 9 days after anthesis. (DOCX) [file pone.0276136.s002.docx]

**
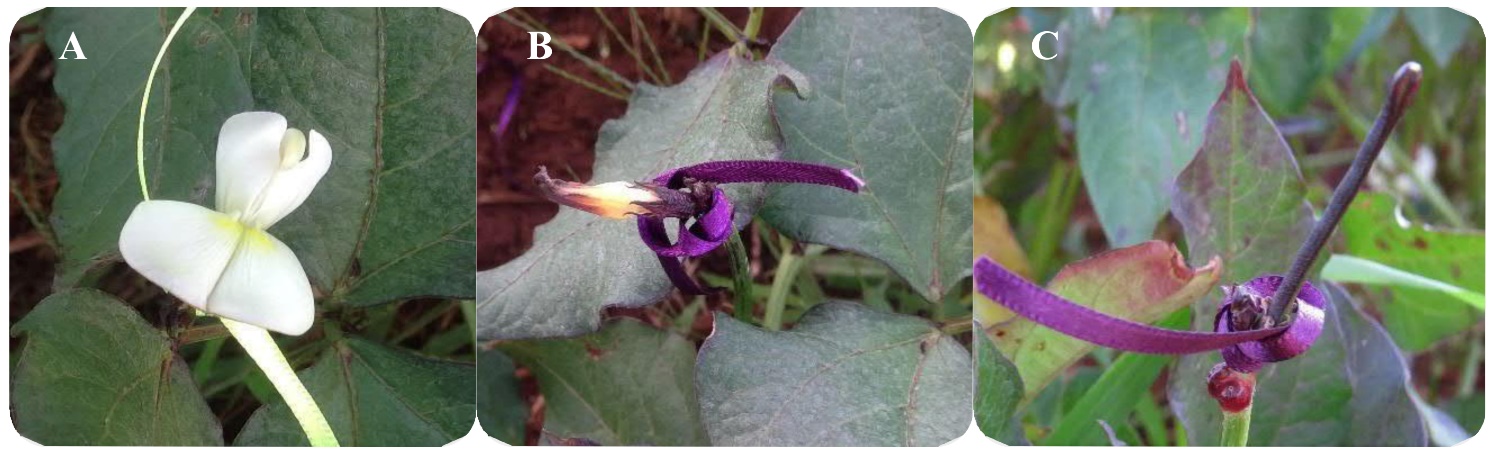
**

**S2 Fig.** Flower tagged and beginning of fruit development of cowpea (*Vigna unguiculata* L., Walp). A: Open flower; B: Flower after 2 days of pollination; C: Fruit in formation 9 days after anthesis (DAA).
