# Supplementary material for: Quality of cowpea seeds: A food security strategy in the tropical environment
Source: PLoS One. 2022 Oct 14;17(10):e0276136. doi: 10.1371/journal.pone.0276136 (PMC9565620; doi:10.1371/journal.pone.0276136)
Supplement: S1 Table — (DOCX) [file pone.0276136.s003.docx]

**S1 Table.** Pre-tests: Information of the data evaluated in cowpea seeds (*Vigna unguiculata* L., Walp) during early maturation stages (17 to 25 DAA).

| DAA ^1^ | WC | DW | G |
| --- | --- | --- | --- |
| 17 | 86.60 | 0.5 | 0.0 |
| 20 | 83.82 | 1.4 | 0.0 |
| 23 | 77.87 | 3.4 | 0.0 |
| 25 | 69.63 | 7.0 | 0.0 |

^1^ DAA: days after anthesis; WC: water content (wet basis, %); DW: dry weight (mg/seed); G: germination capacity (fresh seeds, %).
