# Supplementary material for: Quality of cowpea seeds: A food security strategy in the tropical environment
Source: PLoS One. 2022 Oct 14;17(10):e0276136. doi: 10.1371/journal.pone.0276136 (PMC9565620; doi:10.1371/journal.pone.0276136)
Supplement: Attachment — Submitted filename: Response to Reviewer.doc [file pone.0276136.s004.doc]

Manuscript Number: PONE-D-22-16964

**Quality of cowpea seeds: A food security strategy in the tropical environment**

Alessio Scarafoni PhD,

Academic Editor of PLOS ONE

Dear Dr. Scarafoni,

We thank you and the reviewer for the corrections and suggestions made in the manuscript. Below, we provide our answers point-by-point to the comments and suggestions made.

With warm regards,

Amaral da Silva (corresponding author on behalf of co-authors)

**Reviewer #1:**

**Answer:** The term "theoretical model" has been duly corrected as suggested. Regarding the diagram, the germination rate was added in place of t50. We would like to point out that the images illustrate the cycle of a plant coming from seed with low quality (immature seed) and high physiological quality (mature seed) and not a plant stand. So, if we understand the suggestion, the diagram is adequate to illustrates the proposal of the work. If there are more to correct, we are at your disposal to improve the manuscript.

Detailed comments

**1 and 2. Answer:** As the reviewer suggested, Figures 1, 3, and 5 have been modified. Thanks.

**2. Answer:** The correction has been made (lines 206 and 207). Thanks.

**3. Answer:** The correction has been made (lines 201). Thanks.

**4. Answer:** The correction has been made (line 200). Thanks.

**5. Answer:** The evaluation of normal seedlings at five days made it possible to identify the capacity for complete formation of embryonic tissues early. Only the most vigorous seeds have this capacity. Therefore, we consider that for vigor evaluation, five days was enough to detect what we were looking for. We thank you for the suggestions and we are available for further discussion. Thanks.

**6. Answer:** Yes. The corrections have been made (line 216). Thank you very much.

**7. Answer:** Yes. The corrections have been made (line 217). Thanks.

**8. Answer:** Thank you very much for this observation. The data has been duly reviewed (Figs 1 and 2) and corrections made (lines 208 to 215).

**9. Answer:** All the suggested corrections have been made. Thank you very much (lines 216 to 227).

**10. Answer:** The correction has been made (line 290). Thanks.

**11. Answer:** The correction has been made (line 294). Thanks.

**12. Answer:** The correction has been made (Fig 5). Thanks.

**Reviewer #2:**

**1. Answer:** Based on the data obtained, the constructed diagram allowed us to raise the idea of the use of high quality seeds as a food security strategy. Therefore, we reinforce that the discussion of this topic is based on the last paragraph of the manuscript (lines 296 to 310).

**2. Answer:** The correction has been made. Thanks.

**3. Answer:** We started the conclusion based on the results of the study. Afterwards, we end with a general idea built on these scientific results. We emphasize that the conclusion is not only the concrete expression of the results, but a general interpretation of them with a broader scope. We propose that the seed is the link to food security and has the potential to be used by multi areas in science. Therefore, we consider that our conclusion is correct. However, we are happy to improve it if more corrections are necessary.

**4. Answer:** The correction has been made (line 218). Thanks.

**5. Answer:** The correction has been made (line 218). Thanks.

**6. Answer:** The correction has been made (line 218). Thanks.

**7. Answer:** The correction has been made (line 218). Thanks.

**8. Answer:** The correction has been made (line 218). Thanks.
